# Performance and Robustness of Regional Image Segmentation Driven by Selected Evolutionary and Genetic Algorithms: Study on MR Articular Cartilage Images

**DOI:** 10.3390/s22176335

**Published:** 2022-08-23

**Authors:** Jan Kubicek, Alice Varysova, Martin Cerny, Kristyna Hancarova, David Oczka, Martin Augustynek, Marek Penhaker, Ondrej Prokop, Radomir Scurek

**Affiliations:** 1 Department of Cybernetics and Biomedical Engineering, VŠB—Technical University of Ostrava, 17.listopadu 2172/15, Poruba, 708 00 Ostrava, Czech Republic; 2 MEDIN, a.s., Vlachovicka 619, 592 31 Nove Mesto na Morave, Czech Republic; 3 Department of Security Services, Faculty of Safety Engineering, VŠB—Technical University of Ostrava, ul. Lumirova 3, 700 30 Ostrava, Czech Republic

**Keywords:** medical image segmentation, articular cartilage, regional segmentation, ABC, PSO, DPSO, Otsu thresholding, K-means clustering

## Abstract

The analysis and segmentation of articular cartilage magnetic resonance (MR) images belongs to one of the most commonly routine tasks in diagnostics of the musculoskeletal system of the knee area. Conventional regional segmentation methods, which are based either on the histogram partitioning (e.g., Otsu method) or clustering methods (e.g., K-means), have been frequently used for the task of regional segmentation. Such methods are well known as fast and well working in the environment, where cartilage image features are reliably recognizable. The well-known fact is that the performance of these methods is prone to the image noise and artefacts. In this context, regional segmentation strategies, driven by either genetic algorithms or selected evolutionary computing strategies, have the potential to overcome these traditional methods such as Otsu thresholding or K-means in the context of their performance. These optimization strategies consecutively generate a pyramid of a possible set of histogram thresholds, of which the quality is evaluated by using the fitness function based on Kapur’s entropy maximization to find the most optimal combination of thresholds for articular cartilage segmentation. On the other hand, such optimization strategies are often computationally demanding, which is a limitation of using such methods for a stack of MR images. In this study, we publish a comprehensive analysis of the optimization methods based on fuzzy soft segmentation, driven by artificial bee colony (ABC), particle swarm optimization (PSO), Darwinian particle swarm optimization (DPSO), and a genetic algorithm for an optimal thresholding selection against the routine segmentations Otsu and K-means for analysis and the features extraction of articular cartilage from MR images. This study objectively analyzes the performance of the segmentation strategies upon variable noise with dynamic intensities to report a segmentation’s robustness in various image conditions for a various number of segmentation classes (4, 7, and 10), cartilage features (area, perimeter, and skeleton) extraction preciseness against the routine segmentation strategies, and lastly the computing time, which represents an important factor of segmentation performance. We use the same settings on individual optimization strategies: 100 iterations and 50 population. This study suggests that the combination of fuzzy thresholding with an ABC algorithm gives the best performance in the comparison with other methods as from the view of the segmentation influence of additive dynamic noise influence, also for cartilage features extraction. On the other hand, using genetic algorithms for cartilage segmentation in some cases does not give a good performance. In most cases, the analyzed optimization strategies significantly overcome the routine segmentation methods except for the computing time, which is normally lower for the routine algorithms. We also publish statistical tests of significance, showing differences in the performance of individual optimization strategies against Otsu and K-means method. Lastly, as a part of this study, we publish a software environment, integrating all the methods from this study.

## 1. Introduction

Medical image segmentation represents one of the essential procedures in medical image analysis. The methods, belonging in the area of medical image segmentation, play an important role for the image area decomposition with the focus image understanding. Such methods enable two important issues: (1) the extraction of morphological features of objects of interest and (2) the consequent extraction of various features of such objects with the aim of the quantification of biological tissues [1,2,3,4,5,6,7]. In this context, we are routinely focused on either geometrical parameters of image regions such as the area, perimeter, diameter, or curvature parameters or intensity parameters, including a statistic estimation of the intensity spectrum to quantify the image surface [8,9]. Not having automated image segmentation methods, clinical experts would have to perform medical tissues segmentation manually by contouring objects of interest. Such a procedure would be surely linked with subjective error depended on the skills of the individual physician. On the other hand, manual contouring plays an important role in the objectivization of automated image segmentation methods, where manual annotation normally serves as a gold standard to objectively evaluate the segmentation performance based on selected evaluation parameters such as the index of correlation, the mean squared error (MSE), the structural similarity index (SSIM), and many others [10,11,12,13,14].

Image segmentation includes a lot of methods varying in their mathematical strategy and the aim of the segmentation. Edge detection represents one of the most conventional methods for the automatic contouring of objects of interest. Here, we recognize multiple principles such as the maximum of the first derivative, or the zero crossing detectors [15,16,17]. These methods are normally linked with two main limitations. Firstly, they perform the segmentation of the whole image regardless of the focus on a particular object of interest and mainly they are prone to intensity variations as the impact of image noise, which may significantly influence the segmentation quality. Therefore, these methods are often completed with smoothing filters to improve the segmentation quality. More sophisticated segmentation techniques represent active shape models such as active contours or level set methods, which are capable of focusing on a particular object of interest and within a predefined number of iterations perform the gradual detection of geometrical features of objects of interest. One of the major limitations of these methods is its computing time, because they use a higher number of iterations, and the computing time is also depended on image resolution [18,19].

The most extensive area of the medical image segmentation is regional image segmentation. These methods normally enable medical image decomposition into a predefined number of regions [20,21]. Such a region is perceived as a finite number of image points (pixels or voxels) that mutually share similar features. This predetermines that such regionally oriented methods are able to well recognize biological tissues of interest within individual regions. Regarding the nature of regional segmentation, we recognize so called noninterpreted methods, which normally utilize conventional segmentation strategies and only enable a decomposition of the image points into individual regions without interpreting the content of individual regions [22,23,24]. Nowadays, the recent trends in medical image regional segmentation are mainly focused on so called interpreted methods, enabling the interpretation of individual detected objects. Such methods normally use semantic segmentation [25,26]. Among the benefits of such methods, it is important to mention that these methods require training on huge datasets, which may be a limitation in the context of medical image availability [27,28,29,30].

In this paper, we provide a comprehensive insight in the form of a comparative analysis of selected evolutionary optimization algorithms and genetic algorithms performances, which are used for the tuning of conventional segmentation strategies to achieve a maximal performance under various image conditions and deterioration by image noise. In this study, we compare the performance of optimized segmentation strategies with the elements of artificial intelligence (evolutionary and genetic algorithms) with conventional strategies based on the hard thresholding (Otsu method) and nonhierarchical clustering (K-means). This analysis should objectively point out on the performance and impact of modern optimization methods with artificial intelligence as from the view of segmentation performance and robustness under additive noise and also computing time, which is an important factor of each segmentation procedure regarding complex effectivity. This objectivization analysis investigates the robustness and computing requirements of individual segmentation strategies provided under the influence of various types of additive deterministic noise with dynamical intensity. That enables the study of the performance of individual optimization techniques and its determining parameters along gradually deteriorated conditions and shows the dynamical features of robustness of individual methods. Besides the analysis of performance, we also publish the application of regional segmentation for cartilage features extraction. As a part of our research, we publish a testing software environment, integrating individual methods of regional segmentation with the possibilities to select individual steering segmentation parameters. The software application enables the application of various deterministic noise generators with the settings of the noise parameters, which control noise intensity to simulate image degradation for the testing of individual segmentation strategies robustness. This application also enables the segmentation accuracy evaluation based on selected objectivization parameters, which are also used in this study to evaluate the segmentation performance.

The organization of the paper is following. In Section 2, we provide recent notes and advances in the area of medical image segmentation. In Section 3, we introduce individual segmentation strategies, guidelines for testing of these methods, and datasets of MR images of articular cartilage used in this study. Section 4 is aimed on quantitative results, providing a comprehensive insight on the effectivity, robustness, and limitations of segmentation methods. Section 5 is aimed on complex conclusions, discussion, and future trends of this study.

## 2. Recent Work

In this section, we outline the conventional procedures, serving for a spatial image domain decomposition, which is routinely used as a fundamental tool for medical tissues identification. There are various mathematical approaches, which may be used for the aforementioned image decomposition, including mainly techniques based on histogram partitioning, edge detectors, contours tracing, analysis pixel’s relationships, methods of artificial intelligence, and others [30,31,32,33,34,35,36,37,38].

One of the most popular techniques, and it is also the aim of this paper, is the regional image segmentation bases on the histogram thresholding. These methods normally allow for a histogram decomposition bases on either one, or multiple thresholds, defining individual image regions [37,38]. Here, one of the most popular methods is historically Otsu segmentation, which defines individual thresholds based on the minimization of intra-class intensity variance and the maximization of inter-class variance [39,40,41].

Other popular areas for regional medical image segmentation is clustering analysis. Here, the most popular methods are based on the nonhierarchical clustering such as K-means or fuzzy C-means (FCM) segmentation [42,43]. These methods usually measure distance-based parameters between individual pixels and cluster (region) centroids for the pixel’s classification [44,45]. Such methods are capable of performing image decomposition into various isolated classes based on their features as a level of similarity between the pixel intensity and the centroid [46,47,48,49,50]. Of course, apart from the mentioned approaches, there are plenty of others, which are normally used for medical image segmentation, including edge detectors, outlining image boarders, the methods for consecutive regions forming, such as region growing, or splitting methods, watershed or wavelet transformation, and others [51,52,53]. Of course, in recent times, one of the most popular segmentation methods deals with various applications of machine and deep learning, enabling semantic segmentation instead of the segmentation without interpretation as it is typical in many conventional approaches [54,55,56,57,58,59,60,61,62,63,64,65,66,67,68].

These routine approaches, as we outlined earlier, are usually easily implemented with a reasonable computing time, on the other hand they normally suffer from certain limitations, which may have a significant influence on their effectivity and robustness as well. In the comparison with conventional segmentation methods such as Otsu thresholding, which is based on the hard selection of individual thresholds in histograms, we proposed schemes on thresholding strategies utilizing optimization techniques for optimal thresholds selection from various thresholds combinations. This is supposed to be a more effective approach, which better reflects a pixel’s distribution inside defined regions. Furthermore, we provide analysis of robustness of each tested method upon dynamic various noise influence to objectively show dynamic features of performance when an image domain is gradually deteriorated by additive noise. On the other hand, we are aware that evolution strategies and a genetic algorithm may have a significant influence on the computing time. Therefore, we also publish time complexity analysis, showing their complexity. All the optimized methods we put in a contrast with the conventional approaches such as Otsu thresholding and the K-means method to objectively point out differences in segmentation performance in the context benefits and limitations of using evolutionary and genetic algorithms for various numbers of thresholds and other steering optimization parameters of these optimization techniques.

## 3. Materials and Methods

Recently, various implementations of evolutionary and genetic optimization strategies have increased in popularity for solving various engineering problems in the area of optimal settings of steering parameters of various procedures. The main aim of this paper is to objectively point out on the effectivity and robustness (a level of stability in various image environment) of selected thresholding-based regional segmentation strategies, being optimized with evolutionary computing methods (ABC and PSO with its variants: FPSO (fuzzy particle swarm optimization) and DPSO (Darwinian particle swarm optimization)) and genetic algorithms as from the view of their effectivity of segmentation, features extraction, and also time complexity. On the other hand, ABC algorithm is used in the combination with fuzzy thresholding, which forms individual segmentation regions based on the membership functions for each region, where pixels are classified into regions based on the membership values as we describe further. This is also an important issue of this study as shows the impact of fuzzy thresholding besides conventional hard thresholding histogram partitioning.

In the contrast of the optimized methods, we put two selected conventional segmentation approaches, which have been considered for a long time as standards for medical image segmentation, as they were used in plenty of research studies, dealing with various object detections from medical images. The first one is Otsu thresholding, which is the implementation of so-called hard histogram thresholding, and the second method is K-means, which defines segmented regions based on the nonhierarchical clustering. Implementation of these routine segmentation strategies, which utilize various principles for image segmentation, point out on differences in performance between non-hierarchical clustering and thresholding for regional image segmentation. In a general way, we define the optimization problem of a set of thresholds:

(1) $$T=\left\{T_{1},\;T_{2},\ldots ,T_{n}\right\}$$

In such configuration, we search for an optimal combination of individual thresholds ($T_{1},\;T_{2},\ldots ,T_{n}$), defining individual segmented regions, which the best satisfies optimization criteria, which in evolutionary and genetic algorithms is given by fitness function, which is described further. As the definition of fitness function, we use a measure of entropy (Kapur entropy), which well defines pixel’s distributions in segmented regions.

### 3.1. Segmentation Methods

In this section, we introduce individual segmentation strategies. Here, we describe the evolutionary strategies based on the ABC and PSO (and its variants) and genetic algorithms for histogram-based thresholding. In contrast with these strategies, we put the conventional methods based on the hard thresholding (Otsu thresholding) and K-means, which classifies pixels into regions based on a similarity (Euclidean distance) with region’s centroid. These conventional methods do not contain any optimization elements, so it would be interesting to compare the performance of optimization strategies with these segmentation routines.

In order to objectively report the effectivity and robustness of individual methods, we employ selected deterministic noise generators with dynamic noise intensity (controlled by their steering parameters). Dynamical noise impact is manifested by gradual deterioration and modification of pixel’s intensity distribution, which supposedly should have impact on the segmentation robustness as we provide modeling in the section results. In order to provide such analysis, we employ Gaussian, salt and pepper, speckle, and Rician noise generators with dynamical range of their noise impact.

**Figure 1:**
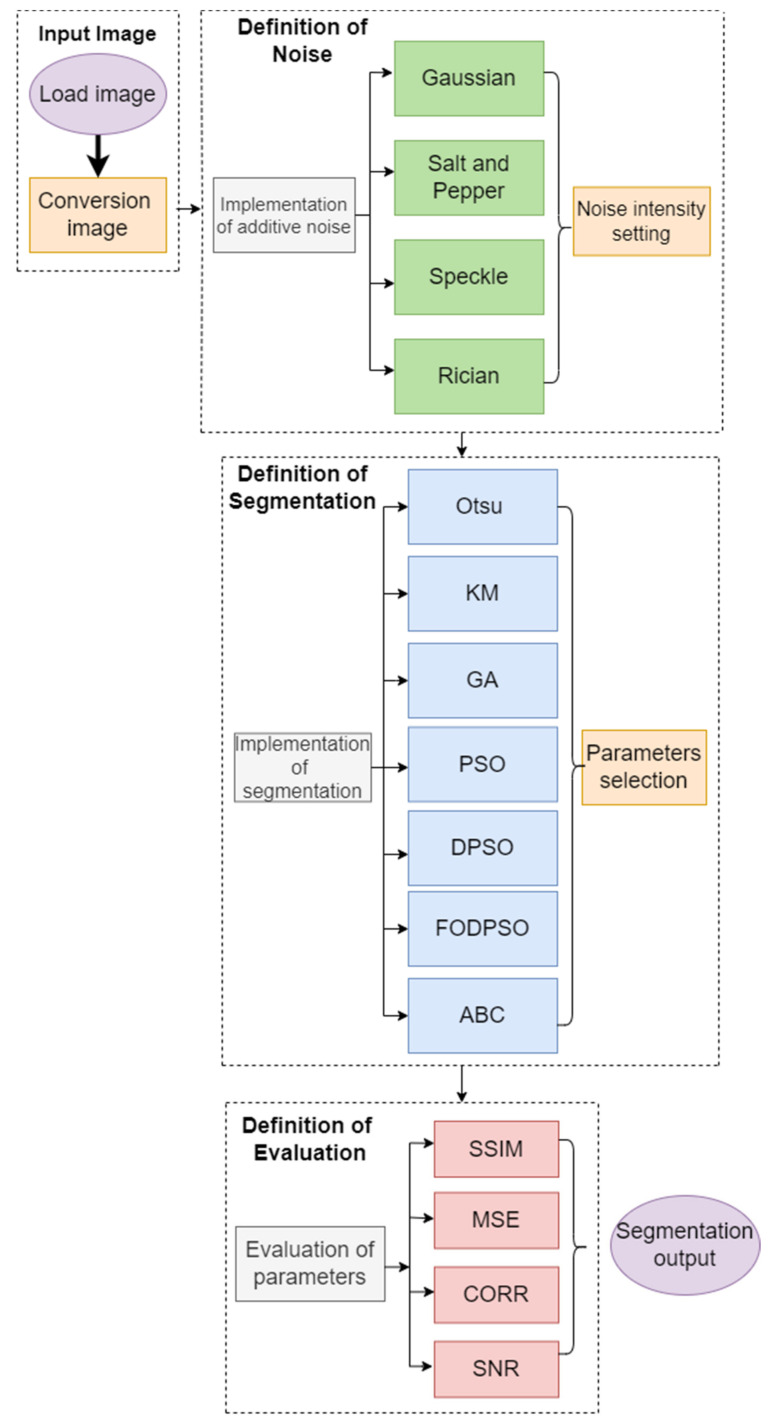
Complex general flowchart of testing environment for segmentation evaluation.

In order to objectively measure the noise impact of the segmentation performance of individual studied methods, we employ selected evaluation parameters, which are focused on measuring a level of similarity or difference between the native segmentation (with a zero level of added noise) and respective noise level. This approach enables objective performance evaluation of studied segmentation strategies. For this analysis we use the following objectivization parameters: structural similarity (SSIM), mean squared error (MSE), correlation coefficient (CORR), and signal noise ratio (SNR). Figure 1 represents a whole testing environment, which is the main aim of this paper, including application of noise generators, segmentation strategies, and parameters for evaluation.

#### 3.1.1. Otsu Thresholding

Otsu method [66] is one of the sophisticated thresholding methods, which is based on the number of regions selected, Otsu method algorithm determines the optimal thresholds according to the histogram. Image segmentation uses the classification of pixels into segmentation regions. The basis of the method is the statistical parameter of variance, which characterizes the variability of individual pixels in the image. The criteria for this classification include minimizing the intra-class variance or maximizing the inter-class variance. Otsu method uses histogram thresholding to define the number of regions. It searches for the segmentation class with the smallest variance, i.e., the optimally chosen threshold. This technique is classified as a statistical method because it works based on the statistical parameter of variance, which characterizes the variability of the distribution of individual pixels in the image. The equation for calculation of the within-class variance at any threshold is:

(2) $$\sigma^{2}\left(t\right)={\;\omega }_{\mathrm{bg}}\left(T\right)\sigma_{\mathrm{bg}}^{2}\left(T\right)+\omega_{\mathrm{fg}}\left(T\right)\sigma_{\mathrm{fg}}^{2}\left(T\right),$$

where $\omega_{\mathrm{bg}}\left(t\right)$ and $\omega_{\mathrm{fg}}\left(t\right)$ are the probability of number of pixels for each class at threshold T and $\sigma^{2}$ is variance of color values (pixels). The variance is represented by equation:

(3) $$\sigma^{2}\left(t\right)=\frac{\sum {\left(x_{i}-\bar{x}\right)}^{2}}{N-1},$$

where $x_{i}$ presents pixel value in group of bg or fg and $\bar{x}$ presents the mean pixel value in group of bg or fg and N represents the number of pixels.

$\omega_{\mathrm{bg}}$ and $\omega_{\mathrm{fg}}$ are calculated by:

(4) $$\omega_{\mathrm{bg}}=\frac{P_{\mathrm{BG}}\left(T\right)}{P_{\mathrm{all}}},$$

(5) $$\omega_{\mathrm{fg}}=\frac{P_{\mathrm{FG}}\left(T\right)}{P_{\mathrm{all}}},$$

where P_BG_ is count of background pixels at threshold T, P_FG_ is count of foreground pixels at threshold T, and P_all_ is the total count of pixels in image.

Otsu method can be extended for a multiregional segmentation scheme [67], where more thresholds are defined. In this configuration, the image histogram is divided into equal areas, and for each such area the own threshold is defined. Finally, the original image is segmented by using all these thresholds. Supposing we have *L* image intensities in the range: $\left[0,\;1,\;2,\ldots ,\;L\right]$ and the parameter *p* stands for the number of thresholds. A width of one area is defined as the ratio:

(6) $$a=\frac{L}{p}$$

Optimal thresholds (*P*) for individual areas are defined as the maximization of inter-class variance:

(7) $$P_{p}=max_{p}\left(\sigma^{2}\left(t\right)\right)$$

#### 3.1.2. K-Means

K-means method [68] is classified as a non-hierarchical clustering method. K-means method allows assigning individual pixels into segmentation classes to which they belong based on distance. It uses nonhierarchical clustering for pixels assignment and searches for the minimum distance between the pixel and the selected center of gravity (centroid). A given pixel is then assigned to the region to which it has the smallest distance. The most commonly used metric is Euclidean, which measures the distance of pixels in the feature space according to the following equation:

(8) $$D(\vec{x},\vec{y})=\sqrt{\sum_{i=1}^{n}{\left(x_{i}-y_{i}\right)}^{2}},$$

where $\vec{x},\vec{y}$ are the feature vectors, $D(\vec{x},\vec{y})$ is the resulting distance, *n* is the dimension of the space, *x* and *y* are the pixel coordinates.

Pixel *x*_*i*_ is assigned to class *y*_*i*_ according to the following relation:

(9) $$y_{i}=\arg min_{j}\left\|x_{i}-\mu_{j}\right\|$$

The following vector recalculation calculates the new values of the vectors *μ*_*j*_ as the mean values from the pixels *x*_*i*_ that were classified into the class determined by the vector *μ*_*j*_. The new value of *μ*_*j*_ is calculated according to equation:

(10) $$\mu_{j}=\frac{1}{n_{j}}\;\sum_{i=1,y_{1}=j}^{n}\left(x_{i}\right),\;$$

where *n*_*j*_ denotes the number of pixels and *x*_*i*_ classified in the second step into the class determined by the vector *μ*_*j*_. The vector classification and recalculation steps are repeated until at least one vector *x*_*i*_ is classified into a different class than it was classified in the previous step. The pixel in each class that possesses the maximum frequency is determined as the centroid. The disadvantage of this method is that by assigning objects to each class, it can only determine whether the object belongs to the cluster or not. Therefore, K-means is classified as a hard approach technique.

#### 3.1.3. ABC Evolutionary Optimization

ABC algorithm or artificial bee colony is an algorithm that belongs to the algorithms based on the swarming behavior of animals. Specifically, it is the behavior of bees looking for food. The principle of the algorithm is that it tries to provide the best approximate solution with low computational requirements. The bees function as a whole in a certain way and the allocation of the different roles in the community is automatic. These are the employed bees (EB), the onlooker bees (OB), and the scouts (SB) whose task is to improve the overall food resources. The basic parameters of ABC include the number of food sources, the limit, and the number of iterations. The limit determines after how many iterations a worker will abandon their solution if they have not been able to improve it.

The total population (SN) consists of an equal number of EBs and OBs, with each EB having one temporary solution *R*_*i*_ adjacent to the solution *X_i_*. Parameter *X_i_* = {*X_i_*, 1, *X_i_*, 2, …, *X_i_*, *p*} represents the i-th solution in the swarm of bees, where p represents the number of parameters that are optimized. In the first stage of the algorithm, these two solutions are compared using the fitness function and if the solution of the fitness function *R*_*i*_ is better, it is kept as the new solution from this pair. Otherwise, no change occurs. This is done for each of the pairs *X_i_* and *R*_*i*_. It is necessary to apply a selection limit *L*_*v*_ specifying the maximum number of attempts in selecting a solution *X_i_* in case an optimal solution *R*_*i*_ cannot be found.

However, if an optimal solution cannot be found even after exhausting *L*_*v*_, we consider it as a burnt-out solution. The second stage is essentially an extension of EB. It works with OB in which the existing individual solutions are tested from different perspectives. The more optimal solution should have a higher value of *P*_*i*_. After selecting the food source *X_i_*, a neighboring food source *R*_*i*_ is determined and their fitness values are compared. The last part is the scouts searching for new food sources instead of depleted sources. The process of evaluating solutions is iterative, in most cases 100 cycles are applied. The output of this optimization method is the set of all admissible solutions, where in the final step the solution possessing the maximum value of *P*_*i*_ is selected.

#### 3.1.4. PSO Evolutionary Optimization

PSO, or particle swarm optimization, is an evolutionary optimization computing technique inspired by the social behavior of birds and fish swarms. This method uses a population of particles that fly in an irregular motion through a given space at a certain speed. The position of each agent is given by the vector *x*_*i*_, and its movement corresponds to the velocity *v*_*i*_. The particle velocity is determined as follows:

*v*_*i*_(*t*) = *v*_*i*_(*t* − 1) + *c*_1_ ∗ *rand*_1_(*p*_*i*_ − *x*_*i*_(*t* − 1)) + *c*_2_ ∗ *rand*_2_ (*p*_*g*_ − *x*_*i*_(*t* − 1)), (11)

where *c*_1_ and *c*_2_ are positive numbers, *rand*_1_ and *rand*_2_ denote random numbers from the range 0 to 1. Equation is composed of three parts. The inertia and attraction to the best-found position of a given particle *p*_*i*_, and we denote the value of the fitness function at this position by *p*_*best*_. This attraction is multiplied by a random weight *c*_1_ ∗ *rand*_1_ and is called the memory of the particle. The third part of the equation is the attraction to the best-found position of the particle *p*_*g*_, and we denote the corresponding fitness value by *g*_*best*_. The aforementioned attraction is again multiplied by a random weight *c*_2_ ∗ *rand*_2_ and is called shared information, or also shared knowledge. Each individual remembers their previous best value and the best value of their neighbors. The agents therefore use the information from the best particle, and therefore this algorithm is more memory efficient than the genetic algorithm.

#### 3.1.5. FPSO Evolutionary Optimization

FPSO stands for fuzzy particle swarm optimization. It is a modified PSO algorithm using fuzzy logic theory. The position and velocity of the particles in this algorithm are defined to represent the relationship between the fuzzy and the variables. Fuzzy logic controller with two inputs and one output improves the performance of PSO. The two input variables represent current best performance evaluation (CBPE) and current inertia weight. The output variable is change in inertia weight. CBPE needs to be normalized according to the following formula:

(12) $$\mathrm{NCBPE}=\frac{\mathrm{CBPE}-{\mathrm{CBPE}}_{\min}}{{\mathrm{CBPE}}_{\max}-{\mathrm{CBPE}}_{\min}},$$

where CBPE_min_ is the true minimum and CBPE_max_ represents the suboptimal CBPE. CBPE normalization is used to make the algorithm applicable to a wide range of optimization processes. A non-optimal CBPE is considered to be any solution with a CBPE greater than or equal to CBPE_max_. These fuzzy variables are defined as fuzzy sets with nine rules for a fuzzy system.

#### 3.1.6. DPSO Evolutionary Optimization

DPSO is a Darwinian algorithm extending the PSO algorithm by natural selection and survival of the fittest to increase the ability to escape from local optima. Darwinian particle swarm optimization (DPSO) allows many swarms of test solutions to exist at any point in time. Each swarm works like a regular PSO algorithm, except that it uses natural selection (Darwinian survival of the fittest) to enhance the ability to escape from a local optimum.

The principle of the solution search is that if it is heading towards a local optimum, then the search for a solution in that region is terminated, and the search for another area begins. Each swarm is monitored at every step. If swarms improve, they are rewarded. The reward is to extend the lifetime of the particles or to produce offspring. If swarms stagnate, they are punished. The punishment consists of shortening the lifetime of the swarm or removing particles. After the removing the particle, instead of being set to zero, the counter is reset to a value approaching the threshold number, according to:

(13) $${\mathrm{SC}}_{C}\left(N_{\mathrm{kill}}\right)={\;\mathrm{SC}}_{C}^{\max}\left[1-\frac{1}{N_{\mathrm{kill}+1}}\right],$$

where N_kill_ represents a number of deleted particles from the swarm, ${\mathrm{SC}}_{C}^{\max}$ represents that the maximum number of swarms must not be exceeded. Whereas the new swarm is created with a probability based on the equation:

(14) $$p=\frac{f}{NS},$$

where *p* is the probability, *f* is a random number in the interval 0 to 1, *NS* represents the number of swarms. Each swarm is evaluated using the fitness function of all particles. In this way, it is possible to analyze the overall state of the swarms separately and thus update the neighborhood and individual best positions of each particle. New particles are created if a new global solution is found. Conversely, particle extinction occurs if the swarm does not find a more suitable state in a defined number of steps.

#### 3.1.7. Genetic Algorithms-Based Optimization

Genetic algorithms are based on natural processes with gradual elimination and subsequent selection of the most suitable solutions. It is a combination between biology and mathematics. Patterns from living nature are used, which initially work by chance and gradually produce better solutions. These patterns are then applied using a mathematical model to a variety of technical applications, including image processing using segmentation techniques.

Genetic algorithms use special procedures to find the optimal solution using selection, crossover, and mutation operations. They start with random selection and search for new, better solutions; the most optimal solution is then selected from the results. All algorithms include a fitness function that provides information about the quality of the solution. Each approach has specific parameters that must be set. Examples are the number of regions, the number of initialization solutions, the parameters of the fitness function, or the possible number of iterations, which means how many steps the algorithm will take to find the best solution. For GA, the number of crossovers from each iteration, the so-called crossover, is also set. At the beginning, the structure of an individual is proposed to express its quality and to make the crossover and subsequent procedures easier. This process is known as initialization.

The selection operator is used to select individuals for further reproduction, it copies the selected strings from the old generation to the new one. Mathematically, it can be expressed as follows:

(15) $$p_{i}=\frac{f_{i}}{\sum_{1}^{N}f_{i}},$$

where p_i_ is the probability of selecting the *i*-th individual from the population and f_i_ corresponds to the fitness value of the *i*-th individual. The crossover operator is analogous to biological reproductive crossbreeding and can therefore be described as the combination of two parents producing an offspring.

The final stage in the creation of a new generation is mutation, which also plays an important role. It ensures diversity and increases the probability of escaping from the local optimum. It can discover a trait in each generation that no individual has had before and therefore could not pass on to its offspring.

#### 3.1.8. Application of Optimization Strategies for Image Segmentation

In a general way, we define the search for optimal combination of thresholds to separate pixels into predefined number of segmentation regions. In order to do this task, we must specify the criteria, based on which, any optimization strategy recognizes an optimal configuration of segmentation model. This task is performed by using fitness function (fit), which is capable of evaluating each possible solution within optimization strategy. In our study, we use a unified version of fitness function to all the techniques were comparable. We define the fitness function based on the Kapur entropy [69,70]. By this way, we can compute and quantify an amount of information, which is presented in each segmented region. We take advantage of this fact to maximize Kapur entropy measure to obtain the segmented regions, containing concentrated intensity distributions without outlying values, causing inhomogeneities. Based on this optimization definition, we define a set of Kapur entropies ($H_{0},\;H_{1},\ldots ,H_{n}$) for *n* regions, defined by the set of thresholds $T_{1},\;T_{2},\ldots ,T_{n}$ in the following form:

(16) $$H\left(T_{1},\;T_{2},\ldots ,T_{n}\right)=\overset{n}{\underset{i=0}{\sum }}H_{i}$$

In this configuration, we compute Kapur entropy for each region by the following way:

(17) $$H_{0}=-\overset{T_{1}-1}{\underset{j=0}{\sum }}\frac{p_{j}}{\omega_{0}}ln\left(\frac{p_{j}}{\omega_{0}}\right),\;\omega_{0}=\overset{T_{1}-1}{\underset{j=0}{\sum }}p_{j}$$

(18) $$H_{1}=-\overset{T_{2}-1}{\underset{j=T_{1}}{\sum }}\frac{p_{j}}{\omega_{1}}ln\left(\frac{p_{j}}{\omega_{1}}\right),\;\omega_{1}=\overset{T_{2}-1}{\underset{j=T_{1}}{\sum }}p_{j}$$

(19) $$H_{n}=-\overset{L-1}{\underset{j=T_{n}}{\sum }}\frac{p_{j}}{\omega_{n}}ln\left(\frac{p_{j}}{\omega_{n}}\right),\;\omega_{n}=\overset{L-1}{\underset{j=T_{n}}{\sum }}p_{j}$$

In these equations (Equations (15)–(17)), the parameters $\omega_{0},\;\omega_{1},\ldots ,\omega_{n}$ stand for the probabilities for each segmentation class. Based on the definition of Kapur entropy, we build the fitness function (Equations (18)), which quantifies Kapur entropy to recognize worse and better threshold selection, where we suppose the best threshold configuration maximizes the fitness function.

(20) $$fit\left(T_{1},\;T_{2},\ldots ,T_{n}\right)=\arg max\left\{H\left(T_{1},\;T_{2},\ldots ,T_{n}\right)\right\}$$

**Figure 2:**
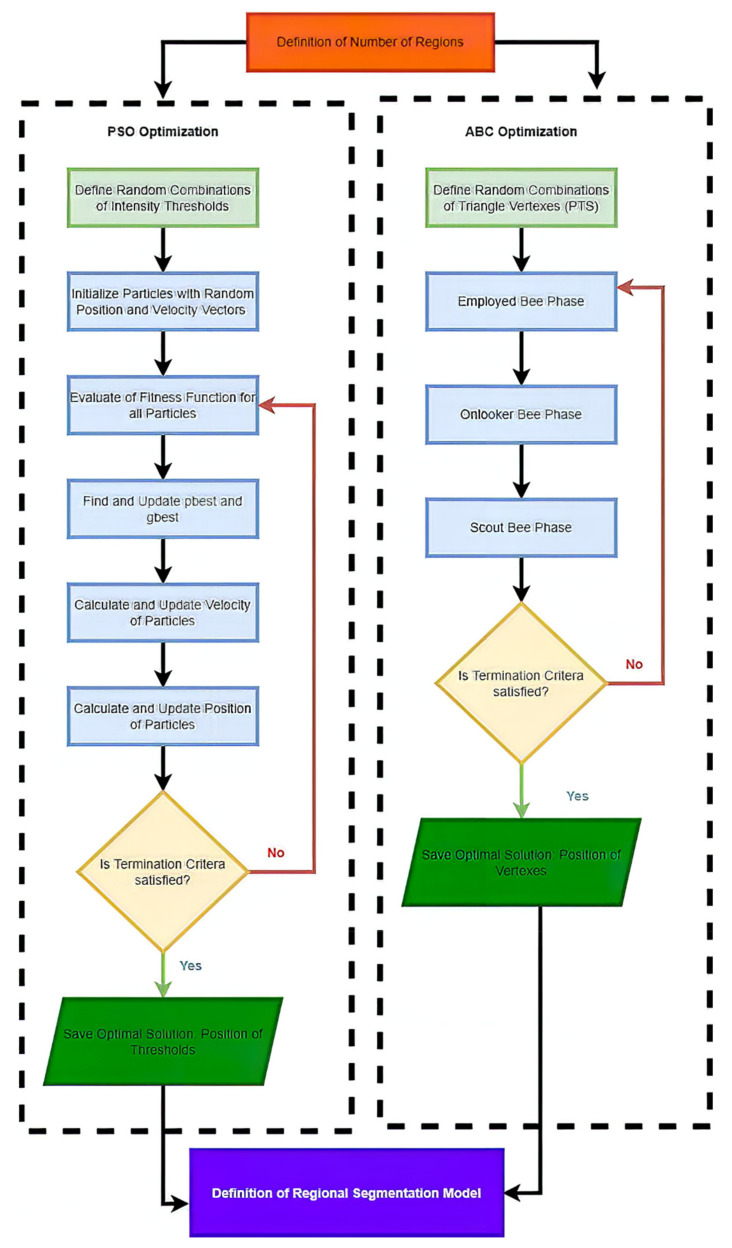
Example of PSO and ABC algorithms for definition of optimized concepts of hard and soft thresholding.

In the following text, we summarize the basic concept of soft thresholding for medical image segmentation. To justify the situation of the application of the evolution algorithms for hard and soft thresholding, we provide the example of usage PSO algorithm (Figure 2) for the definition of hard thresholding-based segmentation and ABC algorithm, which is, in this study, used for the optimization of an optimal location of vertexes of membership functions in soft thresholding, as we describe further in the text.

Supposing we have a monochromatic image with 8-bit depth (256 gray intensities), which may be described by the intensity function $I\left(x,y\right)$, where *I* stands for the monochromatic image and x,y represents the pixels’ coordinates in 2D image raster. To simplify this image definition, we use the notation *I*(*r*) for the image definition. Supposing that *I*(*r*) should be decomposed into *L* regions, which should be extracted with using of soft thresholding by the way to obtain the segmented image, denoted as *M*(*r*) with the definition:

(21) $$M\left(r\right)=g_{s}\left\{I\left(r\right)\right\},$$

where *g_s_*{.} stands for a segmentation method (soft thresholding), which may be considered as a function, providing mapping *N_I_* levels of monochromatic image into *L* regions, that means: *g_s_*: *N_I_* → *L*, where *L* < *N_I_*. After performing the segmentation, is it supposed that each pixel *I*(*r*) is given by a membership level in each of *L* regions. In this definition, the soft thresholding utilizes fuzzy triangular membership functions, which assign a membership level for each pixel in each fuzzy-based region. We use the notation: *µ_l_*(*x*), *l* = *1*, …, *L* for the fuzzy membership function in *l*th region.

The soft thresholding particularly utilizes a pseudo trapezoid-shaped (PTS) function. This function may be defined directly based on the centroid’s definition. These centroids could be calculated by using clustering analyses, where the image data would be decomposed into regions, and the centroids of such regions would represent triangle vertex. Unfortunately, such definition would not reflect the pixel’s distribution inside the regions and only rely on clustering performance. Therefore, we employ the ABC algorithm to search for an optimal distribution of these triangle vertex of PTS to find an optimal segmentation model. As we defined earlier in the ABC definition, the parameter: *X_i_* = {*X_i_*, 1, *X_i_*, 2, …, *X_i_*, *p*} defines ith possible solution for bee swarm. Here, this parameter particularly represents a set of random combinations of triangle vertexes to be optimized using ABC optimization. Besides the definition of the vertexes, we define the further conditions (rules) for the construction of PTS functions:

- Complete division: $\forall x,\;\exists \mu_{l}\left(x\right),\;1\leq l\leq L$, so that $\mu_{l}\left(x\right)>0$
- Consistency: $if\;\mu_{l}\left(x_{0}\right)=1$, then $\mu_{k}\left(x_{0}\right)=0,\;\forall k\neq l$
- Normality: $\max\left(\mu_{l}\left(x\right)\right)=1$

**Figure 3:**
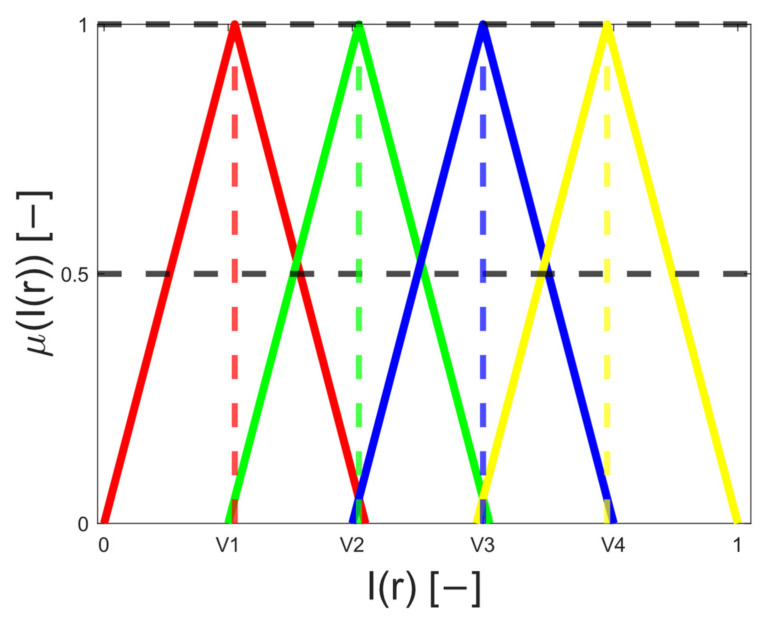
Definition of sequence of four PTS functions for the soft thresholding-base segmentation model (L=4).

As the example (Figure 3), we provide a sequence of four vertexes (V_1_–V_4_), which defines four segmented regions.

The membership function of the pixel *r* from the image *I*(*r*) in the *l*th segmentation class is given by the expression $\mu_{l}\left(I\left(r\right)\right)$. By using of the PTS function, we have: $\overset{L}{\underset{l=1}{\sum }}\mu_{l}\left(I\left(r\right)\right)=1$. This method basically provided transformation of the image pixels into the fuzzy space, where each pixel is described with using of all the membership functions (segmentation model with *L* regions) by the way:

(22) $$\mu \left(I\left(r\right)\right)=\left[\mu_{1}\;\left(I\left(r\right)\right)\;\mu_{2}\;\left(I\left(r\right)\right)\ldots \;\;\mu_{L}\;\left(I\left(r\right)\right)\right]$$

In this definition with PTS functions, only two adjacent membership functions give non-zero membership levels for each pixel. Based on this definition of fuzzy space, we define defuzzification to finally classify the pixels into individual classes based on the maximal level of membership functions.

(23) $$M\left(r\right)=argmax_{l}\left\{\mu_{l}\left(I\left(r\right)\right)\right\}$$

Here, the expression $M\left(r\right)$ stands for the segmented image base on the soft thresholding.

### 3.2. MR Datasets of Articular Cartilage

In this section, we describe the datasets that we use for the testing of the analyzed segmentation methods. Since the musculoskeletal system plays one of the most important roles in the human body, ensuring movement, we are focused on the MR images of articular cartilage from various MR sequences, which are crucial for movement. The musculoskeletal system in general contains various structures thatenable persons to move. In the main principle, this system can be divided into muscles, which serve as the executors of motion, and the bone supporting system with joints, ligaments, and tendons. Besides the motion activities, this system also perform the further important tasks, including upright posture, protective function of vital organs, and functions, ensuring communication such as the contraction of mimic muscles and gesticulation.

Among other medical imaging methods, magnetic resonance plays an important and indispensable role in investigating tendon and muscle traumas and disorders with the aim to distinguish ganglia, cysts, hematomas, and early degenerative structural changes of articular cartilages. Here, the most common MR techniques utilize T1 (longitudinal) and T2 (transverse) relaxation times. In the examination of articular cartilage, routine procedures including a spin-echo sequence are used. Individual tissues have various T1 and T2 relaxation times. Therefore, it makes differences in MR signal strength, which reflect measurable differences in grayscale intensities. Here, darker tissues are perceived as hyposignal, contrarily lighter tissues are hypersignal. Based on this fact the obtained MR images allow T1 and T2 weighting. This fact is frequently utilized in articular cartilage imaging with the aim to distinguish the healthy structure from pathological disorders such as early stage of cartilage loss in osteoarthritis.

In our study, we use three variable sequences of articular cartilage, including T2-weighted images, proton density-weighted images, and gradient echo images. For each mentioned group, we tested in total of 1000 MR images from the public database Osteoarthritis Initiative (OAI) [71,72] to provide a robust comparison among individual segmentation approaches.

In the basic principle, the fat saturation MR sequence involves firstly the excitation and consequently dephasing of the spinning protons in fat tissues. This process is driven by the application of a lipid-specific radiofrequency pulse. This pulse is applied before each repetition of each 2D or 3D sequence. A significant benefit of this approach is a substantial contrast between non-lipid and lipid tissues.

**Figure 4:**
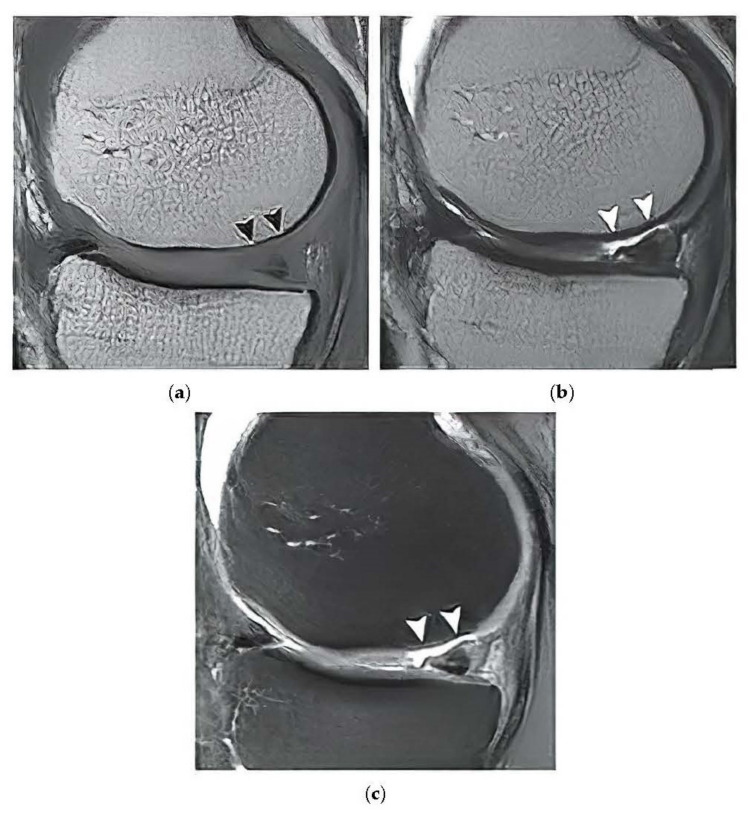
Example of sagittal 2D fast SE image, which was acquired based on the various MR sequences from the same knee’s area: (**a**) T1-weighted image shows a weak contrast between synovial fluid and cartilage surface. A significant limitation is not providing the information for a proper assessment of focal cartilage defect (arrows), (**b**,**c**): T2-weighted image (**b**) and weighted proton density (**c**), image provides a better contrast between the cartilage surface and synovial fluid, enabling the identification of a full cartilage defect (tip of arrow) in medial femoral condyle.

The proton density-weighted MR imaging is able to recognize in contrast the cartilage defects and abnormal cartilage composition in their tissues. This imaging sequence enables a suitable investigation of the cartilage morphology, ligaments, and menisci. The fat-saturated proton density-weighted images are suitable for the investigation of a low-signal intensities, which is a typical case of early cartilage loss. Therefore, this technique is well suited for the examination of osteoarthritis. As an example of the articular cartilage data, we provide a comparison of various sequences from the same cartilage area (Figure 4).

**Figure 5:**
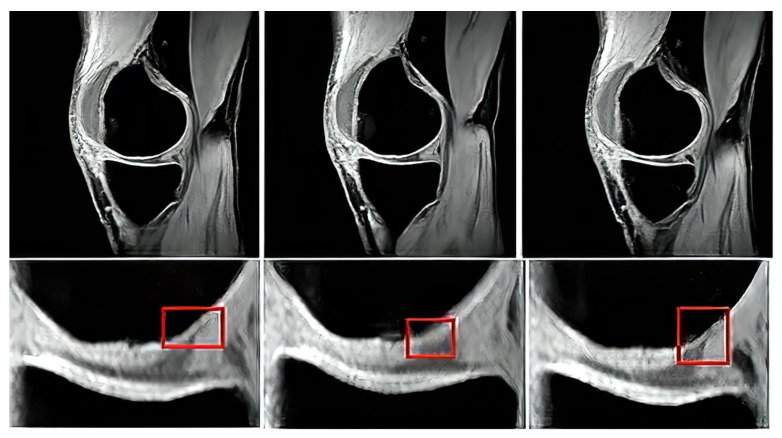
Example of proton-dense sequence for investigation of articular cartilage with early cartilage loss. The first row shows a sequence of three native images from the MR dataset and the second row represents image RoIs, focusing on cartilage area, where the red squares point out MR signal change in cartilage structure that indicate the early cartilage loss.

Proton density sequence plays an indispensable role in structural investigation of the early stage of articular cartilage loss. Here, only a weak contrast between a common cartilage surface and such pathological findings are notable. Therefore, for our analysis these data are substantially important. To objectivize such findings, we provide the example (Figure 5) from our dataset, where such investigations can be observed.

In order to objectivize the acquisition parameters for the tested images, we provide Table 1, which summarizes the important acquisition parameters for individual MR techniques for articular cartilage imaging, used in this study. Here, we mainly indicate the parameters such as FOV, matrix size, acquisition time slice thickness, interslice gap, scanning mode, and findings. We use the unified settings of matrix size and FOV (Table 1) to all the segmentation methods and were objectively comparable in the same dimension of image domain, which is important for performance comparison of individual segmentation strategies as well as their time complexity, which should be compared for the same image size.

### 3.3. Deterministic Artificial Noise Generators

In order to provide the analysis of robustness of individual segmentation techniques, we employ various image noise generators, which simulate gradual deterioration of spatial image area by using their steering parameters, as we describe further. For our analysis, we use the following noise generators: Gaussian noise, speckle noise, salt and pepper noise, and Rician noise.

#### 3.3.1. Gaussian Noise

Gaussian noise represents white statistical noise. This type of noise is due to natural sources such as ambient temperature. The distribution of Gaussian noise is uniform in the image and affects all pixels with the same intensity. It is a normal distribution of noise distribution in the image. Gaussian noise can be defined using the following formula:

(24) $$G\left(x\right)=\frac{1}{\sigma \sqrt{2\pi }}e^{\frac{{\left(x-\mu \right)}^{2}}{2\sigma^{2}}},$$

where *x* represents the luminance of the noise, *σ^2^* is the variance, and *μ* represents the mean.

#### 3.3.2. Speckle Noise

Speckle noise is a common noise that occurs in all coherent imaging systems (lasers, acoustic systems, ultrasound). The cause of this noise is the interference of a signal that has a different phase when returning from the target. This noise is displayed in the image as dark pixels with a higher brightness value. The input parameter is the speckle noise variance. Speckle noise can be described by the formulation:

(25) J=I+n∗I,

where *I* presents the input image, *J* is the noise distribution in the input image, and *n* presents unified zero mean value of the noise in input image.

#### 3.3.3. Salt and Pepper Noise

Salt and pepper represents impulse noise. The image degradation takes place at several pixels in the image, with the pixel carrying no information about the original value. The pixel values in the image are replaced by values of 255 or 0. Thus, this noise is represented in the image as white and black dots resembling salt and pepper. The input parameter for setting the noise is the density. This noise is most often noticeable during data transmission.

#### 3.3.4. Rician Noise

Rician noise represents the most typical noise in images taken by magnetic resonance. Rician noise is based on Gaussian noise in that the real and imaginary parts of the signal are corrupted by an uncorrelated zero mean. The magnitude of Rician noise can be expressed using the following formula:

(26) $$M=\sqrt{{\left(I+n_{1}\right)}^{2}+n_{2}^{2}},$$

where *M* represents the signal magnitude, *I* represents the original image with negligible noise intensity, and $n_{1}$ and $n_{2}$ are Gaussian noise variables with zero mean and equal variance $\sigma_{n}^{2}$. Here, we can define the probability density function (PDF) for an image, which is corrupted by Rician distribution by the following way:

(27) $$p\left(M|I,\sigma_{n}^{2}\right)=\frac{M}{\sigma_{n}^{2}}exp\left(-\frac{M^{2}+I^{2}}{2\sigma_{n}^{2}}\right)I_{0}\left(\frac{IM}{\sigma_{n}^{2}}\right)u\left(M\right)$$

In this definition, $I_{0}\left(.\right)$ depicts the 0th order of modified Bessel function of the first kind, and the parameter *u*(.) stands for Heaviside step function [56].

### 3.4. Application and Settings of Noise Generators

Firstly, we introduce the settings of noise deterministic generators, which are used in this study. Each of the noise generators is determined by its steering parameters, which determine the noise intensity. We use a gradual ascended sequence of the noise intensities to effectively simulate the segmentation performance degradation upon increasing the level of the noise-based image deterioration. In the Gaussian noise (G), we use a constant dispersion ($\sigma^{2}=0.01$) and variable mean value of the noise (μ), in the salt and pepper (SaP) noise we provide testing for variable noise density (*d*) and for speckle (Sp) and Rician noise (Ric), we control the noise intensity via the parameter variance ($\sigma^{2}$). For the purposes of testing, we use 20 noise levels to simulate the segmentation performance (Table 2).

**Figure 6:**
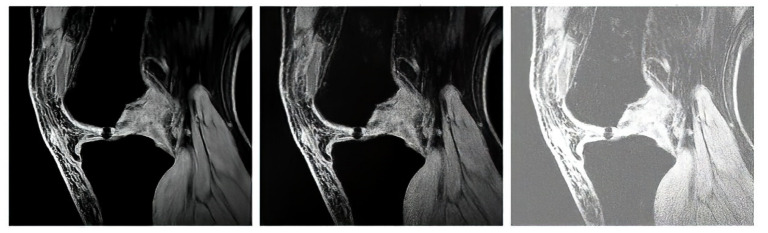
Application of Gaussian noise on native MR cartilage image with intensities (from left): native image, $\sigma^{2}=0.01$, μ = {0.005, 0.01}.

**Figure 7:**
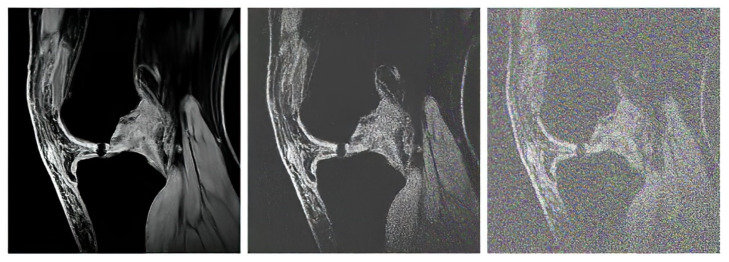
Application of Rician noise on native MR cartilage image with intensities (from left): native image, $\sigma^{2}=\left\{0.2,\;0.4\right\}$.

**Figure 8:**
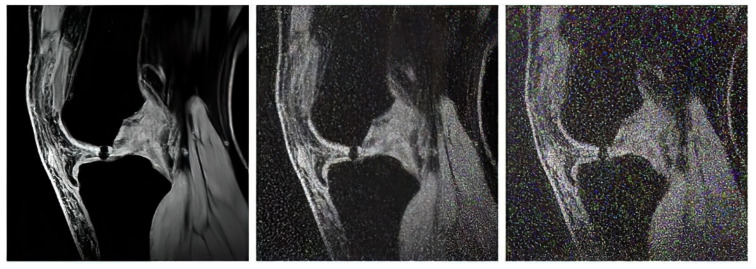
Application of salt and pepper noise on native MR cartilage image with intensities (from left): native image, $d=\left\{0.2,\;0.4\right\}$.

**Figure 9:**
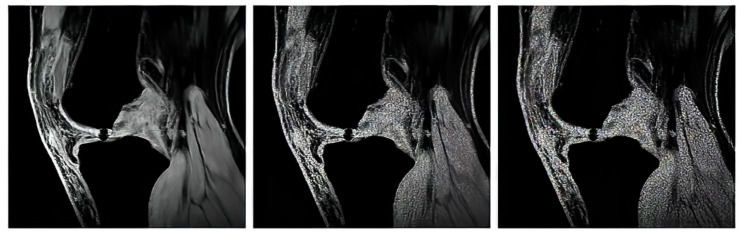
Application of speckle noise on native MR cartilage image with intensities (from left): native image, $\sigma^{2}=\left\{0.2,\;0.4\right\}$.

We gradually applied the noise generators with the range of the noise intensity parameters to artificially simulate the noise impact on the pixel’s distribution. As we stated earlier, for each noise, we set 20 levels on the noise intensity. That means each native MR image contains in total 21 images for testing of segmentation algorithms (1 native image + 20 noise levels). For each image, we defined a multidimensional array, where all these noisy images are stored. In the following outputs: Figure 6, Figure 7, Figure 8 and Figure 9, we provide the examples of gradual deterioration of individual noise generators, which we used for the testing.

### 3.5. Evaluation Parameters

All the performance characteristics are constructed by the way we use the segmentation of native MR images as a gold standard against individual segmentation in individual noise levels. This approach enables objective measurement of the noise influence for each noise level. This finally shows dynamical features of performance within continuous degradation by image noise with variable intensity. The following parameters are considered for this study.

SSIM or structural similarity index [59] is a parameter that allows us to objectively express the similarity of two images *x* and *y* using a metric. This parameter is defined by the following formula:

(28) $$\mathrm{SSIM}\;\left(x,y\right)=\frac{\left(2\mu_{x}\mu_{y}+C_{1}\right)\left(2\sigma_{xy}+C_{2}\right)}{\left(\mu_{x}^{2}+\mu_{y}^{2}+C_{1}\right)\left(\sigma_{x}^{2}\sigma_{y}^{2}C_{2}\right)},$$

where *C_i_* = ${\left(k,\;l\right)}^{2}$, where *l* represents the dynamic range of pixel values, *k* << 1 are small constants with values usually 0.02, *μ* represents the weighted average of the *x* and *y* images, and *σ* represents the covariance of *x* and *y*. These components in the formula allow to compare between *x* and *y* images: brightness (*l*), contrast (*c*), and texture (*s*). The comparison method extracts structural information from the scene. This parameter takes values from −1 to 1, with 1 representing the absolute match between the *x* and *y* images. Here, we consider that *x* represents the segmentation with zero level of additive noise and *y* represents the segmentation output with respective level of the noise.

Correlation coefficient represents the linear correlation between two images *x* and *y*. The correlation coefficient is defined as the ratio of the covariance of the variables *x* and *y* multiplied by their standard deviations. The Pearson pairwise correlation coefficient *r* can be expressed using the following equation:

(29) $$r=\frac{\sum \left(x_{i}-\bar{x}\right)\cdot \left(y_{i}-\bar{y}\right)}{\left(n-1\right)s_{x}s_{y}},$$

where *s_x_* and *s_y_* represent standard deviations and $\bar{x}$ and $\bar{y}$ represent the arithmetic mean for each of the variables *x* and *y*. The correlation coefficient takes values from −1 to +1, and the closer the absolute value of the correlation coefficient *r* is to one, the closer the relationship between the variables *x*, *y*. The higher the value, the better the segmentation performance.

SNR or signal to noise ratio is a parameter that allows us to express the ratio of useful power to useless power of a signal (image). This parameter is defined by the following equation:

(30) $$\mathrm{SNR}=10\log_{10}\;\cdot \;\frac{\sum_{i=1}^{M}\sum_{j=1}^{N}\left(g_{i,j}^{2}-f_{i,j}^{2}\right)}{\sum_{i=1}^{M}\sum_{j=1}^{N}{\left(g_{i,j}-f_{i,j}\right)}^{2}}$$

SNR or signal to noise ratio is a pair where $g_{i,j}\;$represents the original (gold standard) segmentation (without additive noise) and $f_{i,j}$ is the segmented image with respective additive noise level. The SNR quantity is decibels (dB). SNR values can be interpreted in the form the higher SNR values we achieve for respective segmentation, the better agreement with the gold standard we have, and better segmentation performance we achieve.

MSE, or mean squared error, is a parameter that can be used to objectively evaluate image quality. This parameter expresses the degree of mean squared error between the original image and the segmented image. This parameter is defined by the following formula:

(31) $$\mathrm{MSE}=\frac{1}{MN}\overset{M}{\underset{i=1}{\sum }}\overset{N}{\underset{j=1}{\sum }}{\left(x_{i,j}-y_{i,j}\right)}^{2},$$

where *M* represents the image size in the horizontal direction, *N* represents the image size in the vertical direction, $x_{i,j}\;$corresponds to a pixel in the segmented image at coordinates i and j, and $y_{i,j}$ corresponds to a pixel in the original image at coordinates i and j. For this parameter, the lower the value, the greater the similarity between the images. Practically, we compute the squared differences between the pixels, having the same coordinates in the segmentation matrixes. Consequently, these differences are summed up, and lastly, its mean value is computed. By this way, we compute the mean quadratic difference between the gold standard segmentation and respective noise level segmentation.

The segmentation results (multiregional segmentation) are evaluated via labeling matrix, where each region has a unique number. This number represents an interval of intensity values, which are classified into a specific region. Thus, this can be interpreted as a transformation of a set of intensity values from the image spatial area into the region index. The evaluation parameters reflect pixel reclassification among individual such regions by the influence of additive noise. By adding additive noise with gradually increasing intensity, a respective pixel will have significantly different intensity value when comparing with the situation without additive noise. Therefore, such pixels may be reclassified in a different region. The evaluation of pixel-wise parameters such as SNR or MSE, reflect the impact of change of pixels assignment among individual regions. The main aim of these parameters is reflecting the impact between change of pixel’s assignment in adjacent regions (this is only small change on noise impact) and the shift between more regions, where we can suppose a higher noise impact. This situation firstly reflects the change of pixel region reassignment, but also the shift of pixel intensity by additive noise. Finally, the evaluation parameters quantify the impact of a pixel’s assignment change and thus objectively evaluate a robustness of a respective pixel’s classification upon the image noise with gradual intensity. The higher shift between regions is registrable, the bigger impact on evaluation parameters is, which quantify the performance and robustness of the regional segmentation upon dynamic noise intensity.

## 4. Results

In this section, we introduce quantitative results of testing analyzed thresholding-based segmentation strategies. Here, we provide several types of characteristics to provide an objective view of the segmentation performance and limitations. We provide examples of graphical comparisons of the segmentation methods, which show the influence of the variable image noise of segmentation maps. For the generation of the segmentation maps, we use an artificial color coding. Where each single color represents one region of the segmentation model. To provide a complex view on the segmentation performance, we provide this testing for a variable number of segmentation classes because this parameter has a substantial effect on the segmentation performance. One of the important performance features is the time complexity, thus we provide time requirements of individual segmentation methods. This aspect is substantially important when performing a simultaneous segmentation of a stack of MR images. In order to show a statistical significance between the routine methods and optimized segmentation models, we provide the statistical testing of significance of *p*-values for median tests. The last quantitative analysis deals with the extraction of clinically important cartilage features including the area, perimeter, and cartilage skeleton. Here, we show differences of automatic segmentation and the gold standards. Lastly, we provide a presentation of the software environment, which integrates individual reported segmentation strategies with the possibility of selecting steering parameters of segmentation.

### 4.1. Quantitative Segmentation Evaluation

Gradual noise dynamics has a substantial effect on the pixel’s distribution as we mention in the previous examples. In our study, we utilize this fact to test the robustness of segmentation strategies to justify the impact of optimization elements for the performance of regional segmentation as we describe further.

**Figure 10:**
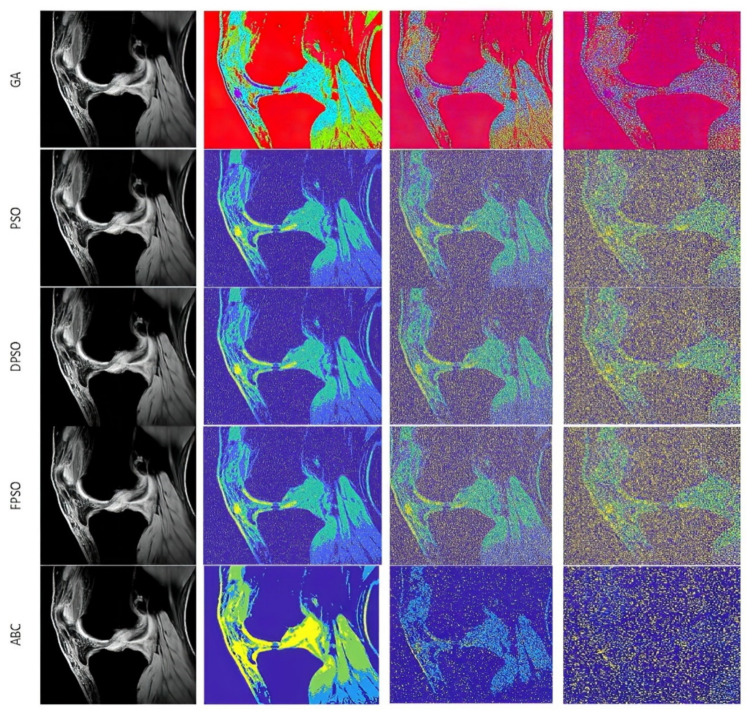
Comparison of regional segmentation models with four regions, which are interpreted by four various single colors for three levels of salt and pepper noise: from left native image and noise density $d=\left\{0.1,\;0.5,\;0.7\right\}$. All the evolution strategies (ABC, PSO, DPSO, and FPSO) have the same settings: 100 iterations and 50 populations.

**Figure 11:**
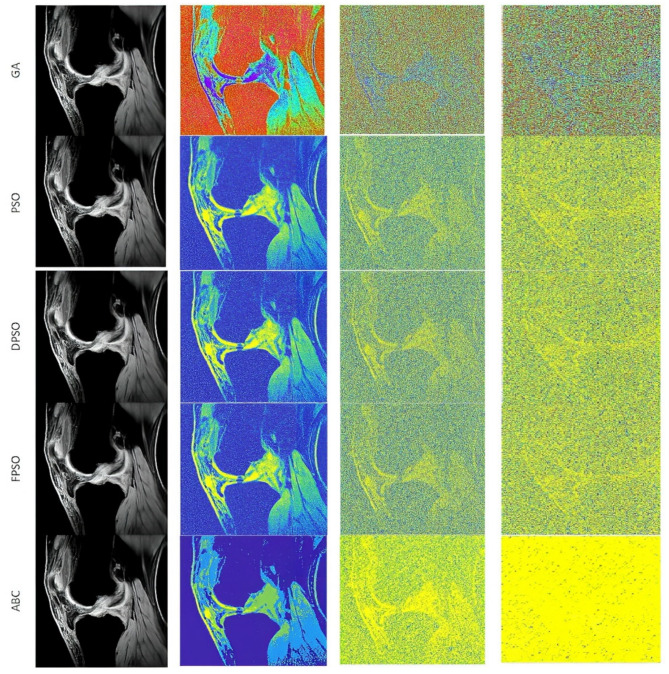
Comparison of regional segmentation models with four regions, which are interpreted by four various single colors for three levels of Rician noise: from left native image and noise density$\;\sigma^{2}=\left\{0.1,\;0.5,\;0.7\right\}$. All the evolution strategies (ABC, PSO, DPSO, and FPSO) have the same settings: 100 iterations and 50 populations.

The first analysis, which we provide is aimed on the graphical evaluation of the analyzed segmentation methods under gradual increasing noise influence. Based on such results, we can subjectively observe clearly visible notable differences in individual methods in segmentation maps. As the example, we provide the comparison (Figure 10 and Figure 11) for all the methods for salt and pepper noise with three levels of density: 0.1, 0.5, and 0.7 and Rician noise with three settings: $\sigma^{2}=\left\{0.1,\;0.5,\;0.7\right\}$.

The segmentation results are interpreted in the form of segmentation maps in the color spectrum (Figure 10 and Figure 11). The interpretation of these color maps is that each segmentation region in the segmentation map is represented by a single value. Thus, the number of colors corresponds with the number of regions. Each such regional model can be interpreted as a transformation of the scale of intensity values to the number of regions. For instance, 8-bit images (256 intensity values) are transformed into four intensities (segmentation model with four regions). The main aim of this analysis is to objectively report how the distribution of a pixel’s assignment into individual regions are modified under the influence of additive noise against the gold standard (segmentation without additive noise influence).

Based on such experimental results, it is noticeable that an increasing noise intensity can significantly impair the quality of the segmentation results. For lower noise levels the segmentation results point out on a good performance, for example the ABC algorithm does not exhibit more significant signs of the noise. On the other hand, higher levels of noise cause significant impairment of the segmentation model consistency. In order to objectively justify this fact, we further provide a robust testing of these segmentation methods based on the mentioned evaluation parameters, to objectively show the change of segmentation performance among individual methods, and also how the number of regions influence the dynamic of segmentation performance. To better justify the testing scheme, we provide testing on routine approaches of Otsu thresholding and K-means clustering. Here, we only set the number of the segmentation regions. Contrarily, in the evolutionary strategies, including ABC, PSO, DPSO, and FPSO, we use a unified number of iterations, 100 (PSO_1_, GA_1_ and ABC_1_) and 500 (PSO_2_, GA_2_ and ABC_2_), and a population size of 50 (PSO_1_, GA_1_ and ABC_1_) and 200 (PSO_2_, GA_2_ and ABC_2_).

**Figure 12:**
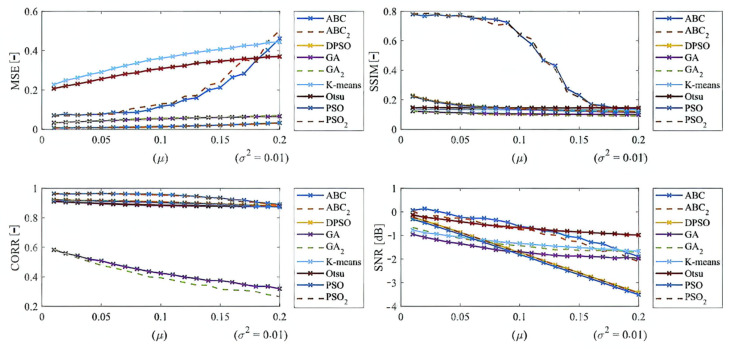
Dynamical features of Gaussian noise influence for regional segmentation effectivity and robustness based on the MSE, SSIM, CORR, and SNR.

**Figure 13:**
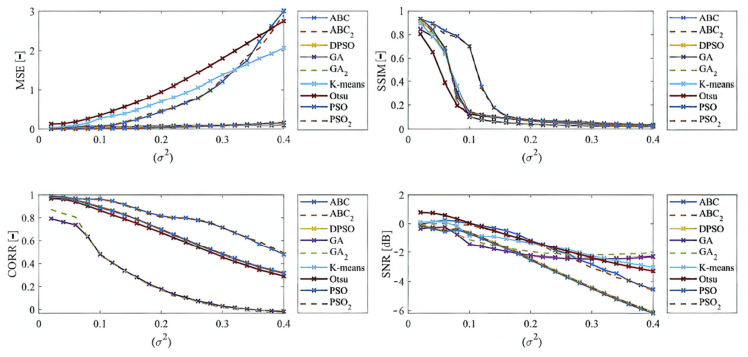
Dynamical features of Rician noise influence for regional segmentation effectivity and robustness based on the MSE, SSIM, CORR, and SNR.

**Figure 14:**
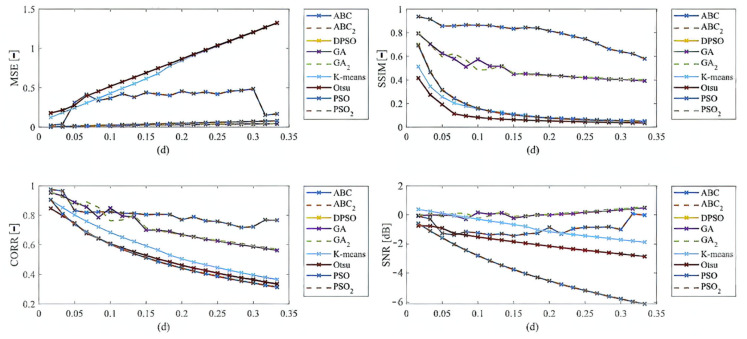
Dynamical features of salt and pepper noise influence for regional segmentation effectivity and robustness based on the MSE, SSIM, CORR, and SNR.

**Figure 15:**
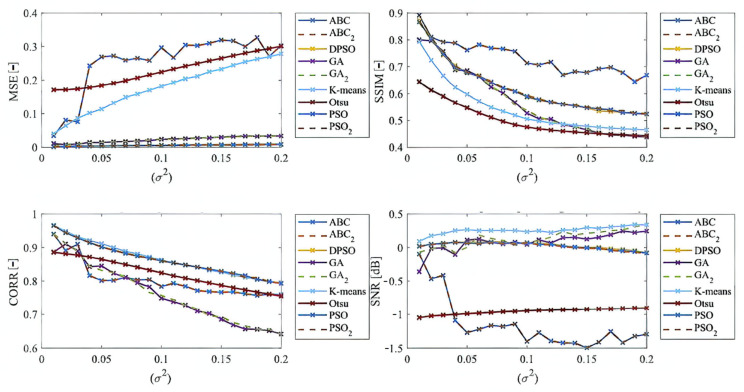
Dynamical features of speckle noise influence for regional segmentation effectivity and robustness based on the MSE, SSIM, CORR, and SNR.

Here, we provide the quantitative comparison of individual optimization techniques for image thresholding-based regional segmentation against selected conventional segmentation approaches, including Otsu hard thresholding and K-means nonhierarchical clustering for regional segmentation. We provide dynamical feature extractions of these methods, reporting effectivity for each noise level and robustness in the form of the trend of the evaluation parameters upon additive noise with dynamic intensity, measured by the mean squared error (MSE), the index of correlation (CORR), the structural similarity index (SSIM), and the signal to noise ratio (SNR). As the example, we provide these characteristics (Figure 12, Figure 13, Figure 14 and Figure 15) for the segmentation models with four regions. The provided characteristics are constructed for 1000 images, where the results for each level of each noise are averaged.

Judging by the experimental results, significant differences in effectivity among individual methods are notable. The trends of the parameters of similarity (SSIM, CORR, and SNR) for Otsu and K-means exhibit significantly lower values when comparing with the evolutionary algorithms. That indicates the notable worse results of these routine algorithms in the comparison with the optimization techniques. The higher these parameters are, the better the performance of respective segmentation is achieved. On the other hand, these routine approaches from the view of MSE exhibit the most rapid increasing trend when comparing with optimization techniques. This is also a sign of the much worse effectivity of Otsu and K-means against the elements of artificial intelligence.

Besides these characteristics, we also publish the averaged results of individual parameters for individual methods. Note that the individual results are average for each parameter and the type of noise. These characteristics (Table 3, Table 4, Table 5 and Table 6) well reflect a global view on the respective segmentation performance. All these comparisons are provided for four segmentation classes. Red values represent the worst results for each test; contrarily, green values represent the best results for each test.

Based on the provided statistical results, we have an insight on a global behavior of the segmentation strategies. Judging by these experimental results, it is notable that the highest performance is achieved in most cases by the ABC algorithm. This is expected due to its enhanced segmentation strategy because it used the fuzzy soft thresholding instead of hard defined thresholds. The further expected fact is the methods with the lowest performance. Here, in most cases are Otsu and K-means, which are comparably worse than the genetic and evolutionary algorithms. The last interesting fact is that PSO and its variants achieve relatively similar results without more significant differences. It is notable that the SNR parameter reports relatively small values. This fact is caused by applying SNR on segmentation maps in the form of index matrixes (for each region we have one unique value) instead of intensity values, where we would have a distribution of 256 intensity levels (in the case of 8-bit images). The MSE parameter achieves small values as well. Here, it is expected, due to the nature of MSE. The lower the MSE is, the higher the agreement between the gold standard and the respective segmentation results we have.

Table 3, Table 4, Table 5 and Table 6 provide the descriptive characteristics of averaged values from all the noise settings for the individual evaluation parameters (SSIM, MSE, CORR, and SNR). These results indicate a better performance of the segmentation strategies, which are optimized with evolutionary or genetic algorithms. On the other hand, these results do not report a statistical significance of testing. Therefore, we further publish statistical testing of the optimized methods against the conventional methods: Otsu thresholding and K-means clustering. Here, we aimed to provide the testing of statistical significance of the mean value for each optimized segmentation strategy against the methods Otsu and K-means for all the evaluation parameters and types of noise. Using paired *t*-tests for mean values assumes that the tested data come from the normal distribution of probability. We tested all the distributions of SSIM, MSE, CORR, and SNR whether they come from the normal distribution. We used the null hypothesis ($H_{0}$) that the data come from the normal distribution and against the alternative hypothesis ($H_{A})$. We place the alternative hypothesis ($H_{A}$), which expresses that the data do not come from the normal distribution: $H_{A}=\neg H_{0}$. For the testing, we use the chi-square test ($\chi^{2}$ test) with the level of significance: α=0.05 and the confidence interval: 1−α=95%. In all the tested parameters SSIM, MSE, CORR, and SNR for the noise: Gaussian, salt and pepper, speckle, and Rician, we found out that the *p*-value is less than α (5%). Therefore, we reject the null hypothesis on the given significance level of 5% that the data come from the normal distribution. Since the data do not come from the normal distribution, we cannot use a paired *t*-test, but we use statistical testing of the median with the Wilcoxon rank sum test. We provide testing for the same significance level: α=0.05 and confidence interval: 1−α=95%. For all the median tests apart from the MSE, we define the null hypothesis as that the median value for the respective evaluation parameter for ABC, GA, PSO, DPSO, or FPSO is greater than the median for Otsu or K-means ($H_{0}:\left(\tilde{*}\right)>\tilde{A},\;\left(\tilde{*}\right)>\tilde{B}$). In the case of MSE, we use the hypothesis: $H_{0}:\left(\tilde{*}\right)<\tilde{A},\;\left(\tilde{*}\right)<\tilde{B}$, here, we supposed that a lower median represents less significant differences in segmentation effectivity. Here, (*) stands for the median value of the respective method and parameter, *A* is the median of Otsu thresholding, and *B* is the median of K-means. Against $H_{0}$, we put the alternative hypothesis as follows: $H_{A}=\neg H_{0}$. In the Table 7, Table 8, Table 9 and Table 10, we present the *p*-values for each test. For each parameter, we report two *p*-values, where the first indicates the test against the Otsu method ($(\tilde{*})>\tilde{A}$ or $(\tilde{*})<\tilde{A}$—for MSE) and the second against K-means ($(\tilde{*})>\tilde{B}$ or $(\tilde{*})<\tilde{B}$—for MSE). The cases when we reject the null hypothesis $\left(p<\alpha \right)$ are indicated as red.

Statistical testing of significance based on the Wilcoxon rank sum test of comparing medians was supposed to declare a level of significance between the respective optimized segmentation strategy and Otsu- or K-means-based segmentation. In order to report this statistical significance, we provide the *p*-values for each test, where its value declares the power of the test (Table 7, Table 8, Table 9 and Table 10). In the context of the employment of genetic algorithms in some cases, we reject the null hypothesis, which means the routine segmentation strategies achieved a significantly higher median. From the global view, in most cases, the evolutionary strategies achieved a higher median than routine algorithms (p-value>α). In these cases, we fail to reject the null hypothesis. The *p*-value also enables measuring the power of the test. The higher it is, the more significant differences we achieved based on the test. In this context, in the case of the ABC algorithm, we normally achieved higher *p*-values in the comparison with other methods. That shows that the combination of fuzzy thresholding with the ABC algorithm appears as the best segmentation strategy in this study.

**Figure 16:**
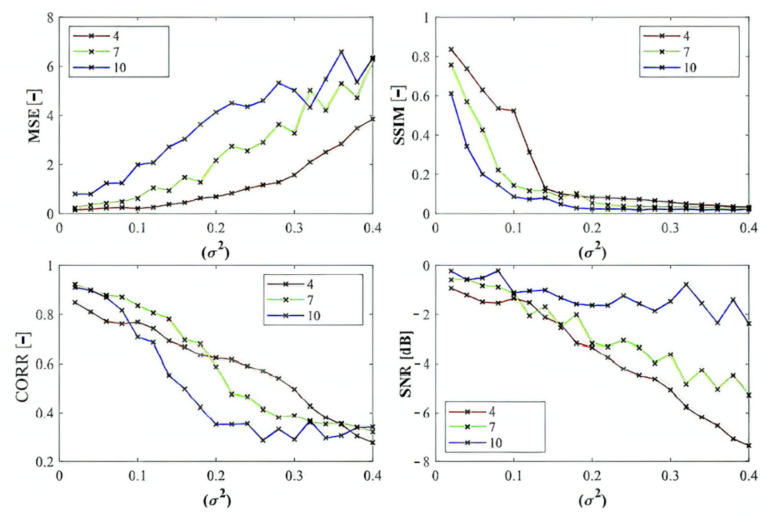
Comparison of various settings of number of regions: 4, 7, and 10 for dynamic influence of Rician noise.

**Figure 17:**
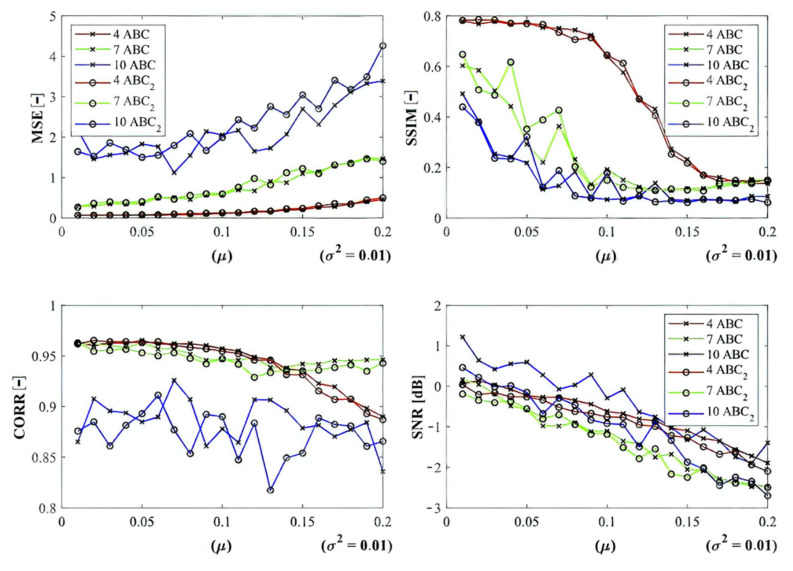
Comparative analysis of ABC algorithm performance for various segmentation settings (4, 7, and 10 regions) for Gaussian noise for two various ABC settings: iterations and population size (ABC_1_, ABC_2_).

In the last part of quantitative comparison, we compare variable numbers of segmentation regions to justify this effect on the segmentation performance. Here, we provide a comparison of 4, 7, and 10 regions of the ABC algorithm for Rician noise (Figure 16). As it is obvious, the number of regions plays an important role in the context of segmentation performance. Based on these results, we can objectively conclude that the segmentation with a lover number of regions mostly achieves a better segmentation performance, contrarily, 10 segmentation regions exhibit the least segmentation performance. The last comparison (Figure 17) that we provide is a detailed insight on the performance of the ABC algorithm with two various settings (ABC_1_, ABC_2_) as we indicated earlier. Here, we show the comparison of these two ABC alternatives for 4, 7, and 10 regions. Furthermore, here, we can observe significant differences in the algorithm’s performance. Mostly, the higher number of regions are set, the worse segmentation results we achieve.

Besides the quantitative characteristics in this section, we also publish a comparison of time requirements. We provide testing (Table 11 and Table 12) of the time complexity for each analyzed method for the simultaneous computing of 100 MR images of articular cartilage (same images were used for all the methods). We provide testing on the following hardware configuration: Intel(R) Core(TM) i5-10300H CPU@2.50 GHz, RAM: 16.0 GB. Based on the achieved results, the significant differences among the methods are notable. Otsu and K-means have the lowest time complexity in all the cases. This is expected due to not requiring optimization techniques. Thus, the main benefit of these methods would be their speed. On the other hand, the use of genetic and optimization algorithms is time demanding, as we declare in the following tables. By this comparison, the slowest method appears to be the genetic algorithm.

### 4.2. Software Environment for Optimized Regional Segmentation Models

**Figure 18:**
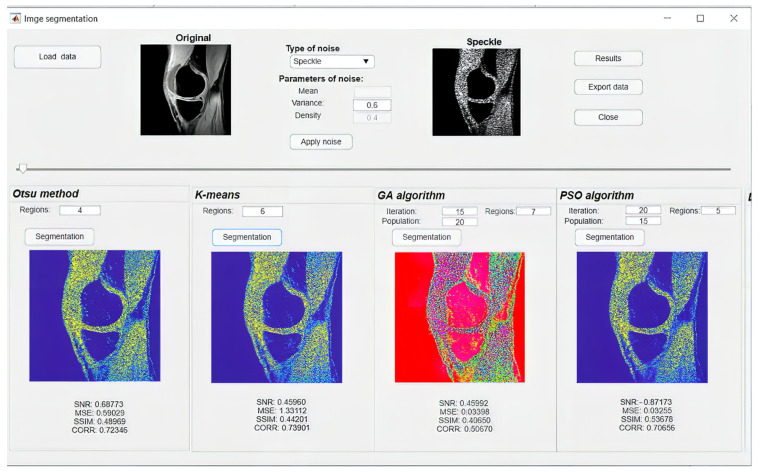
Design of SW environment for testing performance of segmentation algorithms from this study. Here, we show the example of Otsu, K-means, and PSO algorithms with different settings of segmentation under speckle noise influence.

**Figure 19:**
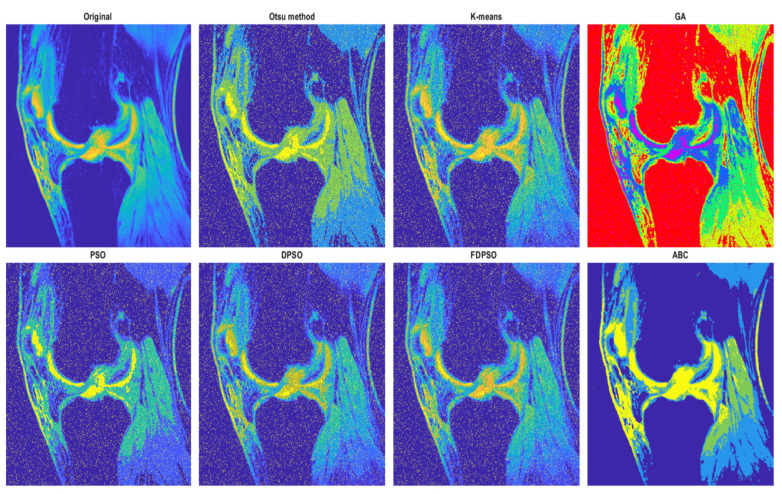
Example of report from the SW for regional segmentation testing. This report contains the segmentation results of all the integrated segmentation techniques under salt and pepper noise with density 0.1.

In this subsection, we introduce the software environment (Figure 18), which is aimed on the testing of the segmentation methods performances. We integrated analyzed segmentation methods into the SW application, providing user-friendly testing of segmentation performance. This software application includes the conventional methods Otsu and K-means, where users can specify the number of regions for the regional segmentation. As the main functionalities, this SW contains procedures for computing segmentation models based on the ABC, PSO, DPSO, FPSO, and genetic algorithms. In these optimization techniques, users are supposed to specify besides the number of regions, the number of iterations and the population size. The SW contains the functions for computing individual noise models, as we introduce in this study, including salt and pepper, speckle, Rician, and Gaussian noise. Users can interactively set the steering parameters for individual noise generators to be consequently applied on the tested image. After applying the segmentation, users will obtain the segmentation performance in the form of list evaluation parameters, including SNR, MSE, SSIM, and CORR. This SW also enables the export of segmentation results (Figure 19).

### 4.3. Clinical Important Features Extraction of Articular Cartilage

**Figure 20:**
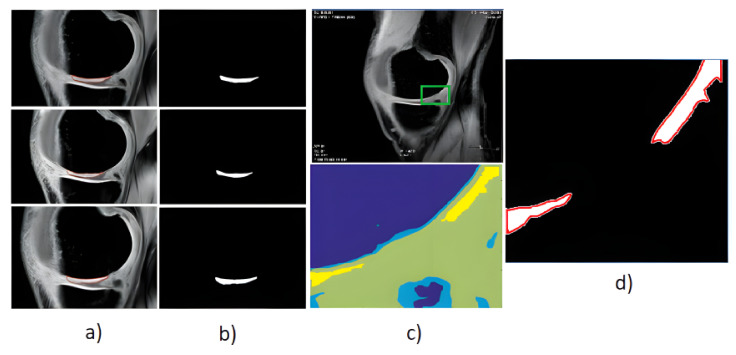
Example of segmentation results for articular cartilage and its features extraction based on fuzzy thresholding with ABC optimization: (**a**) gold standards by manual annotation, (**b**) binary segmentation, (**c**) native MR image with area of interest indicated by the green square (top) and multiregional segmentation with 4 regions (bottom), where yellow contours reflect two lobes of articular cartilage from region of interest, and (**d**) binary extraction of articular cartilage fused with the gold standard (red contour).

Based on the reported analysis of the segmentation performance, mostly the combination of fuzzy thresholding with the ABC evolutionary algorithms appeared as the best segmentation strategy, judging by reported objectivization parameters and mainly provided statistical tests of significance. In this subsection, we would like to provide the last analysis of selected features extraction of articular cartilage from MR images based on the fuzzy thresholding with the ABC algorithm. The aim of this analysis is firstly computing a multiregional segmentation model, allowing for a decomposition of the MR image into a finite number (in this case five) segmentation regions. Consequently, a region, representing the articular cartilage, is selected (Figure 20) as the region of interest, while the rest of the segmentation regions are suppressed from the segmentation model (Figure 20). By this selection scheme, we obtain a binary segmentation model, exclusively classifying the articular cartilage from the rest of the tissues in the MR images. Figure 20 also presents a multiregional segmentation of a part of articular cartilage (femoral cartilage) affected by osteoarthritis of I. grade, which is notable by two segmented lobes of the articular cartilage, and between them is a gap, where the cartilage is missing. To objectize the quality of the articular cartilage extraction and the preciseness of the reported features, we extracted the same features for the gold standard manual segmentation of articular cartilage. Consequently, the feature differences are compared to quantify the segmentation effectivity of articular cartilage detection. Note that we used the following settings for the ABC algorithm: 100 iterations and population size 50. The following cartilage features are considered for evaluation:

- **Cartilage area**—a total count of the pixels, belonging to the model of articular cartilage.
- **Cartilage perimeter**—a perimeter of the cartilage model. Here, we used Sobel edge operator for the detection of cartilage borders, and consequently counted the border pixels.
- **Skeleton of cartilage**—the detection of cartilage skeleton and computing its length.

**Figure 21:**
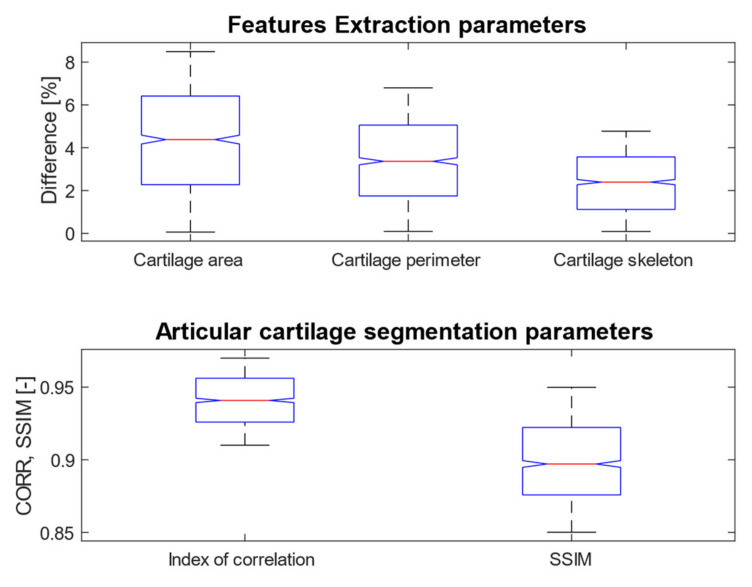
Evaluation of percentage difference distributions for fuzzy soft thresholding with ABC algorithm (against manual segmentation) for cartilage features: area, perimeter, and skeleton and distributions of performance parameters: index of correlation and SSIM.

Based on the segmentation form as binary images, representing extracted articular cartilage and its respective features, we compute descriptive statistics, pointing out on individual distribution’s error functions, which show percentual differences of individual features, and a distribution of values for the individual parameters of segmentation performance (SSIM and index of correlation). Here, the evaluation parameters were computed between the gold standard binary image and the results of the fuzzy thresholding with the ABC algorithm. Figure 21 provides a graphical representation of the distributions of differences for cartilage features and the distribution of values for performance parameters for fuzzy soft thresholding with ABC optimization.

Based on the results of the quantitative analysis of difference function for the extracted features, we did not achieve significant differences between the gold standard images and fuzzy soft thresholding with the ABC algorithm. Mostly the distributions of difference function are kept under 6% of difference. Based on this analysis, we provide the descriptive characteristics (Table 13), which reports the median and standard deviation for each parameter. Based on these results, the best result in median difference is achieved for the feature of skeleton length (2.42%); contrarily, the worst median difference is achieved for the area (4.12%). From the view of measuring variability (standard deviation) of the difference function, the lowest difference is achieved for the skeleton (1.38%) in the contrast with the parameter area, where the difference was the worst (2.44%). The second studied aspect is the performance parameters: the index of correlation and the SSIM. Here, we achieved a higher median for correlation (0.94), where the median for the SSIM was 0.89. Furthermore, from the view of standard deviation, representing the concentration of values is better than the index of correlation (0.017), while in SSIM we achieved 0.028.

## 5. Discussion and Conclusions

Based on the provided results, significant differences among individual methods can be observed. In such comparisons, routine methods show significantly worse results when comparing with the evolutionary algorithms. Along these characteristics, we also provide the average values for all the noise levels to provide a global view on all the studied methods. By this statistical comparison, in most cases the ABC algorithm seems to be the most effective. On the other hand, the use of the genetic algorithm for medical image segmentation strategies does not give satisfactory results. Furthermore, this strategy is enough time demanding. We also studied the time complexity for all the studied methods for three different number of segmentation regions. Here, we can conclude that the increasing number of regions increases the time complexity. These comparisons also bring notable differences among routine methods and optimization strategies. The routine methods are less time demanding in the contrast with the optimization strategies, which is predictable because the evolution strategies usually represent complex procedures. The interesting notable fact from this study is the comparison between the hard thresholding-based approaches with PSO and its variants and soft thresholding with the ABC algorithm. Mostly, soft thresholding overcame the concept of hard thresholding. In this view, the soft thresholding appears as more efficient. On the other hand, the hard thresholding strategies in this study are less time demanding. It is important to mention that the quantitative characteristics of the segmentation performance are represented by the trend characteristics (Figure 14, Figure 15, Figure 16 and Figure 17) of the evaluation parameters, including the index of correlation, SSIM, MSE, and SNR. Ideally, these characteristics would be represented by a monotonous trend clearly defining a progress of the segmentation performance upon dynamic noise. The real results sometimes show certain variations in the form of local oscillations where the complex trends do not have to be always monotonous. This may be caused by the fact that upon various noise intensities, individual segmentation regions are differently affected by additive noise, which contributes to the total effectivity. This phenomenon is connected with the fact that the noise generators work on the principle of random definition of noise.

Although this study reveals a complex view of selected aspects of the employment of evolutionary computing methods and genetic algorithms for medical image segmentation, there are still open issues for further research in this area. In the future research, it would be worth studying in detail various settings of population size and the number of iterations in the context of their impact on the segmentation accuracy. The further important aspect is the definition of criteria for the evaluation of the most suitable threshold settings. Here, we use Kapur entropy as the fitness function. Nevertheless, other alternatives may be plausible. For instance, using a local statistic of variability of pixel’s distribution inside of regions appears to be a reasonable alternative. Based on these open issues we would like to build future research in this area.

## Figures and Tables

**Table 1:** Overview of acquisition parameters for individual MR imaging sequences in this study.

	Fat-Saturated Proton Density-Weighted Sequence	Proton Density-Weighted Sequence	Fat Saturation Sequence
*FOV* (mm)	160 × 160 × 60	160 × 160 × 60	160 × 160 × 60
*Matrix size*	300 × 250	300 × 250	300 × 250
*Acquisition time*	5:60	4:30	2.55
*Slice thickness* (mm)	1.6	1.6	1.6
*Interslice gap* (mm)	0.23	0.15	0.15
*Scanning mode*	2D	2D	2D
*Findings*	Cartilage lesions	Cartilage lesions	Early cartilage osteoarthritis

**Table 2:** Definition of noise generators for segmentation performance analysis.

	Noise Generator			
*Number of Regions*	$G:\;(\sigma^{2}\;=\;0.01$ ), (μ)	SaP: (*d*)	$\mathrm{Sp}:\;(\sigma^{2})$	$\mathrm{Ric}:\;(\sigma^{2})$
4	0.01–0.2	0.17–0.33	0.01–0.2	0.02–0.4
7	0.01–0.2	0.17–0.33	0.01–0.2	0.02–0.4
10	0.01–0.2	0.17–0.33	0.01–0.2	0.02–0.4

**Table 3:** Averaged quantitative results for all the noise levels in Gaussian noise.

Segmentation	*SSIM*	*MSE*	*CORR*	*SNR*
*Otsu*	0.115	0.306	0.889	−0.643
*K-means*	0.104	0.3549	0.884	−1.308
*ABC*	0.515	0.1689	0.944	−0.712
*GA*	0.106	0.0522	0.431	−1.624
*PSO*	0.125	0.0152	0.904	1.894
*DPSO*	0.129	0.0131	0.905	−1.112
*FPSO*	0.128	0.0191	0.871	−1.135

**Table 4:** Averaged quantitative results for all the noise levels in salt and pepper noise.

Segmentation	*SSIM*	*MSE*	*CORR*	*SNR*
*Otsu*	0.0831	0.861	0.556	−0.152
*K-means*	0.113	0.745	0.671	−0.611
*ABC*	0.749	0.211	0.955	1.281
*GA*	0.631	0.0517	0.613	−0.0312
*PSO*	0.141	0.0517	0.633	−2.331
*DPSO*	0.138	0.0526	0.631	−2.266
*FPSO*	0.191	0.0612	0.644	−2.241

**Table 5:** Averaged quantitative results for all the noise levels in speckle noise.

Segmentation	*SSIM*	*MSE*	*CORR*	*SNR*
*Otsu*	0.691	0.204	0.917	−0.675
*K-means*	0.695	0.182	0.911	−0.432
*ABC*	0.744	0.0972	0.955	−0.179
*GA*	0.712	0.0191	0.721	−0.411
*PSO*	0.711	0.0999	0.881	−0.284
*DPSO*	0.711	0.0998	0.884	−0.205
*FPSO*	0.715	0.0995	0.883	−0.201

**Table 6:** Averaged quantitative results for all the noise levels in Rician noise.

Segmentation	*SSIM*	*MSE*	*CORR*	*SNR*
*Otsu*	0.138	1.157	0.645	−1.272
*K-means*	0.168	0.972	0.669	−1.441
*ABC*	0.271	0.0841	0.794	−1.921
*GA*	0.162	0.0701	0.261	−1.822
*PSO*	0.175	0.0501	0.688	−1.288
*DPSO*	0.175	0.0579	0.699	−2.741
*FPSO*	0.114	0.0574	0.645	−2.441

**Table 7:** Results of statistical significance (*p*-values) for individual segmentation strategies against Otsu and K-means segmentation based on Wilcoxon rank sum test for Gaussian noise.

	*p-Value*			
Segmentation	*SSIM*	*MSE*	*CORR*	*SNR*
*ABC*	0.39|0.48	0.35|0.48	0.39|0.44	0.31|0.35
*GA*	0.21|0.29	0.22|0.41	0.04|0.03	0.21|0.28
*PSO*	0.26|0.27	0.31|0.36	0.36|0.36	0.33|0.35
*DPSO*	0.24|0.28	0.29|0.33	0.31|0.33	0.33|0.31
*FPSO*	0.19|0.22	0.33|0.33	0.19|0.28	0.32|0.33

**Table 8:** Results of statistical significance (*p*-values) for individual segmentation strategies against Otsu and K-means segmentation based on Wilcoxon rank sum test for salt and pepper noise.

	*p-Value*			
Segmentation	*SSIM*	*MSE*	*CORR*	*SNR*
*ABC*	0.31|0.42	0.31|0.33	0.39|0.44	0.33|0.35
*GA*	0.22|0.27	0.19|0.18	0.19|0.15	0.15|0.17
*PSO*	0.22|0.29	0.33|0.33	0.20|0.15	0.44|0.41
*DPSO*	0.21|0.26	0.27|0.39	0.19|0.22	0.03|0.04
*FPSO*	0.22|0.24	0.21|0.19	0.14|0.19	0.17|0.16

**Table 9:** Results of statistical significance (*p*-values) for individual segmentation strategies against Otsu and K-means segmentation based on Wilcoxon rank sum test for speckle noise.

	*p-Value*			
Segmentation	*SSIM*	*MSE*	*CORR*	*SNR*
*ABC*	0.37|0.39	0.31|0.27	0.32|0.39	0.31|0.33
*GA*	0.27|0.27	0.19|0.17	0.04|0.03	0.16|0.19
*PSO*	0.23|0.19	0.31|0.33	0.22|0.14	0.32|0.33
*DPSO*	0.23|0.25	0.21|0.29	0.24|0.27	0.21|0.27
*FPSO*	0.27|0.28	0.19|0.21	0.31|0.26	0.18|0.19

**Table 10:** Results of statistical significance (*p*-values) for individual segmentation strategies against Otsu and K-means segmentation based on Wilcoxon rank sum test for Rician noise.

	*p-Value*			
Segmentation	*SSIM*	*MSE*	*CORR*	*SNR*
*ABC*	0.44|0.48	0.48|0.41	0.33|0.49	0.42|0.40
*GA*	0.41|0.39	0.29|0.33	0.01|0.02	0.41|0.37
*PSO*	0.48|0.43	0.37|0.38	0.29|0.29	0.34|0.41
*DPSO*	0.27|0.41	0.35|0.36	0.31|0.42	0.40|0.35
*FPSO*	0.29|0.42	0.33|0.42	0.27|0.33	0.41|0.38

**Table 11:** Analysis of time complexity for native and noisy images for methods Otsu, K-means, and ABC.

Method	*Otsu*			*K-Means*			*ABC*		
Regions	4	7	10	4	7	10	4	7	10
*Native images*	32 s	51 s	76 s	10 s	11 s	12 s	10 min	15 min	20 min
*Gaussian*	27 s	37 s	44 s	4 min	7 min	10 min	191 min	303 min	416 min
*SaP*	26 s	36 s	44 s	3 min	6 min	12 min	191 min	291 min	401 min
*Speckle*	24 s	36 s	41 s	5 min	8 min	12 min	191 min	293 min	398 min
*Rician*	50 s	58 s	63 s	5 min	8 min	17 min	190 min	302 min	408 min

**Table 12:** Analysis of time complexity for native and noisy images for methods GA, PSO, and DPSO.

Method	*GA*			*PSO*			*DPSO*		
Regions	4	7	10	4	7	10	4	7	10
*Native images*	13 m	25 m	35 m	84 s	87 s	96 s	8 m	11 m	11 m
*Gaussian*	280 m	621 m	938 m	26 m	30 m	33 m	178 m	210 m	250 m
*SaP*	394 m	661 m	1027 m	26 m	29 m	33 m	190 m	210 m	254 m
*Speckle*	472 m	740 m	1140 m	27 m	32 m	35 m	191 m	213 m	254 m
*Rician*	558 m	829 m	1239 m	27 m	30 m	33 m	181 m	213 m	254 m

**Table 13:** Descriptive statistic of difference functions for extracted features, where the best results are indicated as green and the worst as red.

Cartilage Features	Median Diff (%)	Standard Deviation Diff (%)
*Area*	4.12	2.44
*Perimeter*	3.51	1.85
*Skeleton*	2.42	1.38

## Data Availability

Data are used from publicly open clinical database *The Osteoarthritis Initiative*. The published SW application for articular cartilage segmentation based on the analyzed segmentation strategies with optimization algorithms can be found via the link: https://www.dropbox.com/sh/mrhnilirfz1tccz/AAATYhbioFPHX9x3cd6gVyUNa?dl=0 (accessed on 20 June 2022).
